# 2,4-Dimethyl­phenyl benzoate

**DOI:** 10.1107/S1600536808017480

**Published:** 2008-06-19

**Authors:** B. Thimme Gowda, Miroslav Tokarčík, Jozef Kožíšek, K. S. Babitha, Hartmut Fuess

**Affiliations:** a Department of Chemistry, Mangalore University, Mangalagangotri 574 199, Mangalore, India; b Faculty of Chemical and Food Technology, Slovak Technical University, Radlinského 9, SK-812 37 Bratislava, Slovak Republic; c Institute of Materials Science, Darmstadt University of Technology, Petersenstrasse 23, D-64287 Darmstadt, Germany

## Abstract

The crystal structure of the title compound (24DMPBA), C_15_H_14_O_2_, resembles those of 4-methyl­phenyl benzoate, 2,3-dimethyl­phenyl benzoate and other aryl benzoates, with similar bond parameters. The central –O—C—O– group in 24DMPBA makes dihedral angles of 85.81 (5) and 5.71 (13)°, respectively, with the benzoyl and phenyl rings, while the two aromatic rings form a dihedral angle of 80.25 (5)°. The mol­ecules are packed with their axes parallel to the *a*-axis direction.

## Related literature

For related literature, see: Gowda *et al.* (2007 ▶, 2008 ▶); Nayak & Gowda (2008 ▶).
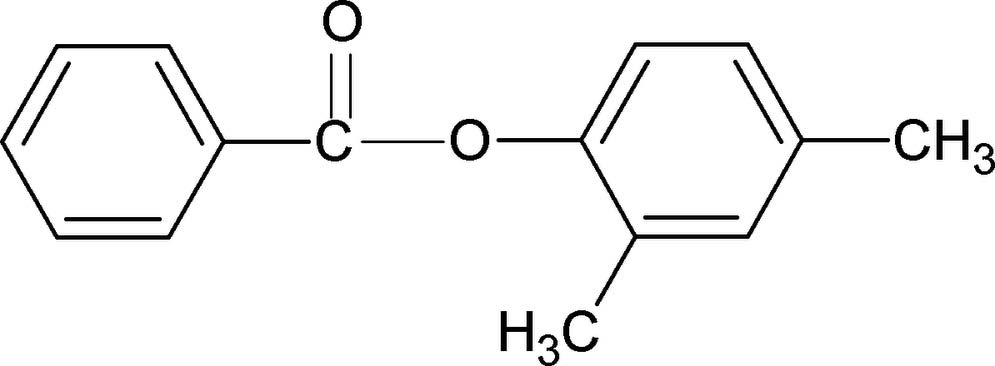


## Experimental

### 

#### Crystal data

C_15_H_14_O_2_
*M* *_r_* = 226.26
Monoclinic, 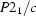

*a* = 7.9813 (2) Å
*b* = 14.3260 (3) Å
*c* = 11.0932 (2) Å
β = 94.028 (2)°
*V* = 1265.26 (5) Å^3^
*Z* = 4
Mo *K*α radiation
μ = 0.08 mm^−1^
*T* = 295 (2) K
0.48 × 0.38 × 0.21 mm

#### Data collection

Oxford Diffraction Xcalibur diffractometer
Absorption correction: multi-scan (*CrysAlis RED*; Oxford Diffraction, 2007 ▶) *T*_min_ = 0.965, *T*_max_ = 0.987
28657 measured reflections
2471 independent reflections
2056 reflections with *I* > 2σ(*I*)
*R*_int_ = 0.031

#### Refinement

*R*[*F* ^2^ > 2σ(*F* ^2^)] = 0.053
*wR*(*F* ^2^) = 0.134
*S* = 1.08
2471 reflections
156 parameters
4 restraints
H-atom parameters constrained
Δρ_max_ = 0.16 e Å^−3^
Δρ_min_ = −0.14 e Å^−3^

### 

Data collection: *CrysAlis CCD* (Oxford Diffraction, 2007 ▶); cell refinement: *CrysAlis RED* (Oxford Diffraction, 2007 ▶); data reduction: *CrysAlis RED*; program(s) used to solve structure: *SHELXS97* (Sheldrick, 2008 ▶); program(s) used to refine structure: *SHELXL97* (Sheldrick, 2008 ▶); molecular graphics: *ORTEP-3* (Farrugia, 1997 ▶) and *DIAMOND* (Brandenburg, 2002 ▶); software used to prepare material for publication: *SHELXL97*, *PLATON* (Spek, 2003 ▶) and *WinGX* (Farrugia, 1999 ▶).

## Supplementary Material

Crystal structure: contains datablocks I, global. DOI: 10.1107/S1600536808017480/ci2614sup1.cif

Structure factors: contains datablocks I. DOI: 10.1107/S1600536808017480/ci2614Isup2.hkl

Additional supplementary materials:  crystallographic information; 3D view; checkCIF report

## Figures and Tables

**Fig. 1.**
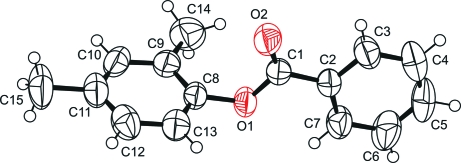
Molecular structure of the title compound, showing the atom labelling scheme. Displacement ellipsoids are drawn at the 50% probability level. H atoms are represented as small spheres of arbitrary radii.

**Fig. 2.**
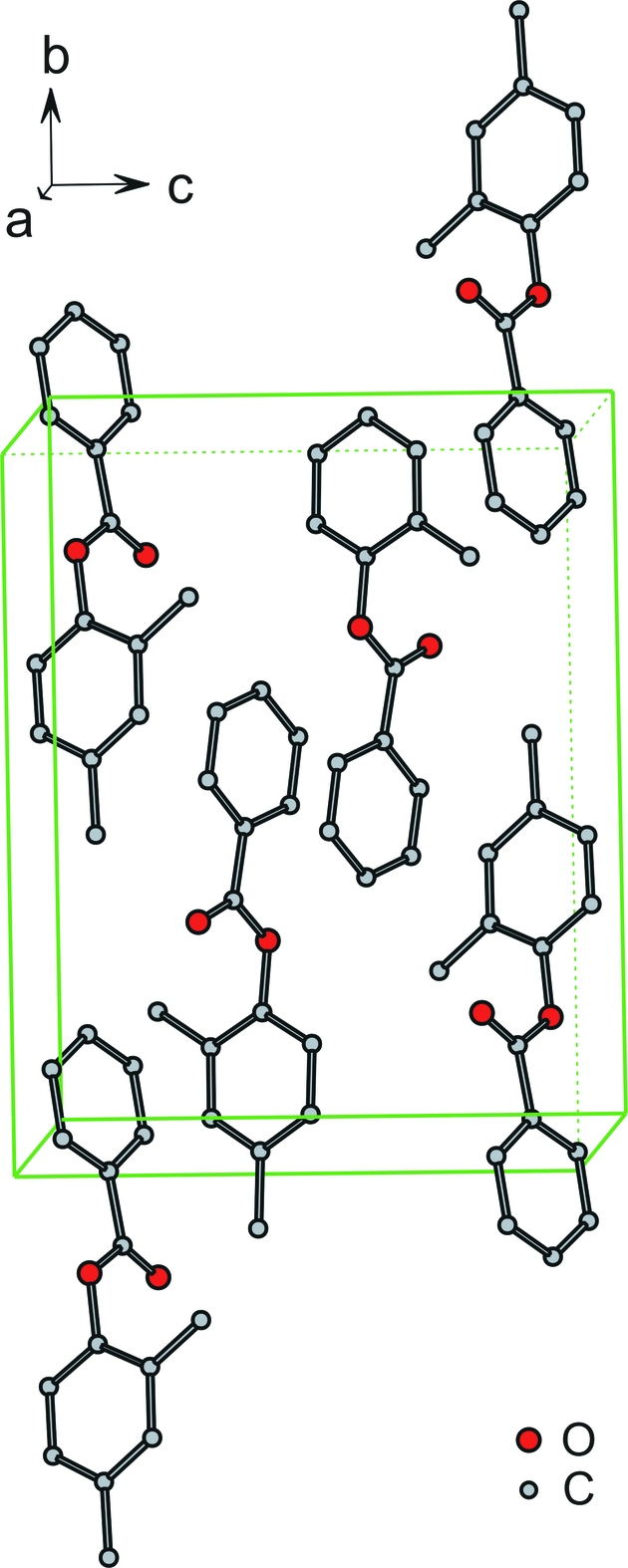
Part of the crystal structure of the title compound as viewed along the a axis. H atoms have been omitted for the sake of clarity.

## supplementary crystallographic information

### Comment

In the present work, as part of a study of the substituent effects on the solid state geometries of aryl benzoates (Gowda *et al.*, 2007, 2008), the structure of 2,4-dimethylphenyl benzoate (24DMPBA) has been determined. The structure of 24DMPBA (Fig. 1) is similar to those of 4-methylphenyl benzoate (4MePBA)(Gowda *et al.*, 2007), 2,3-dimethylphenyl benzoate (23DMPBA) (Gowda *et al.*, 2008) and other aryl benzoates. The central –O—C—O– group in 24DMPBA makes a dihedral angle of 85.81 (5)° with the benzoyl phenyl ring and 5.71 (13)° with the phenyl ring. The two aromatic rings in 24DMPBA form a dihedral angle of 80.25 (5)°. The bond parameters in 24DMPBA are similar to those in 4MePBA, 23DMPBA and other aryl benzoates. Part of the crystal structure of the title compound as viewed along the *a* axis is shown in Fig.2.

### Experimental

The title compound was prepared according to a literature method (Nayak & Gowda, 2008). The purity of the compound was checked by determining its melting point. It was characterized by recording its infrared and NMR spectra (Nayak & Gowda, 2008). Single crystals of the title compound were obtained by slow evaporation of an ethanolic solution and used for X-ray diffraction studies at room temperature.

### Refinement

H atoms were placed in calculated positions and treated as riding with C-H = 0.93Å (aromatic) or 0.96Å (methyl), and *U*_iso_(H) = 1.2 *U*_eq_(CH) and 1.5*U*_eq_(CH_3_). The methyl groups (C14,C15) are disordered over two different orientations. The occupancy factor for the major orientation refined to 0.56 (3) for the C14-methyl group and 0.67 (3) for the C15-methyl group.

### Crystal data

**Table:**

C_15_H_14_O_2_	*F*_000_ = 480
*M**_r_* = 226.26	*D*_x_ = 1.188 Mg m^−^^3^
Monoclinic, *P*2_1_/*c*	Mo *K*α radiation λ = 0.71073 Å
Hall symbol: -P 2ybc	Cell parameters from 12993 reflections
*a* = 7.9813 (2) Å	θ = 3.2–29.3º
*b* = 14.3260 (3) Å	µ = 0.08 mm^−^^1^
*c* = 11.0932 (2) Å	*T* = 295 (2) K
β = 94.028 (2)º	Block, colourless
*V* = 1265.26 (5) Å^3^	0.48 × 0.38 × 0.21 mm
*Z* = 4	

### Data collection

**Table:**

Oxford Diffraction Xcalibur diffractometer	2471 independent reflections
Monochromator: graphite	2056 reflections with *I* > 2σ(*I*)
Detector resolution: 10.434 pixels mm^-1^	*R*_int_ = 0.031
*T* = 295(2) K	θ_max_ = 26.0º
ω scans with κ offsets	θ_min_ = 5.9º
Absorption correction: multi-scan(CrysAlis RED; Oxford Diffraction, 2007)	*h* = −9→9
*T*_min_ = 0.965, *T*_max_ = 0.987	*k* = −17→17
28657 measured reflections	*l* = −13→13

### Refinement

**Table:**

Refinement on *F*^2^	Secondary atom site location: difference Fourier map
Least-squares matrix: full	Hydrogen site location: inferred from neighbouring sites
*R*[*F*^2^ > 2σ(*F*^2^)] = 0.053	H-atom parameters constrained
*wR*(*F*^2^) = 0.134	*w* = 1/[σ^2^(*F*_o_^2^) + (0.0541*P*)^2^ + 0.2483*P*] where *P* = (*F*_o_^2^ + 2*F*_c_^2^)/3
*S* = 1.08	(Δ/σ)_max_ = 0.001
2471 reflections	Δρ_max_ = 0.16 e Å^−^^3^
156 parameters	Δρ_min_ = −0.14 e Å^−^^3^
4 restraints	Extinction correction: none
Primary atom site location: structure-invariant direct methods	

### Special details

**Table:**

Geometry. All e.s.d.'s (except the e.s.d. in the dihedral angle between two l.s. planes) are estimated using the full covariance matrix. The cell e.s.d.'s are taken into account individually in the estimation of e.s.d.'s in distances, angles and torsion angles; correlations between e.s.d.'s in cell parameters are only used when they are defined by crystal symmetry. An approximate (isotropic) treatment of cell e.s.d.'s is used for estimating e.s.d.'s involving l.s. planes.
Refinement. Refinement of *F*^2^ against ALL reflections. The weighted *R*-factor *wR* and goodness of fit *S* are based on *F*^2^, conventional *R*-factors *R* are based on *F*, with *F* set to zero for negative *F*^2^. The threshold expression of *F*^2^ > σ(*F*^2^) is used only for calculating *R*-factors(gt) *etc*. and is not relevant to the choice of reflections for refinement. *R*-factors based on *F*^2^ are statistically about twice as large as those based on *F*, and *R*- factors based on ALL data will be even larger.

### Fractional atomic coordinates and isotropic or equivalent isotropic displacement parameters (Å^2^)

**Table:**

	*x*	*y*	*z*	*U*_iso_*/*U*_eq_	Occ. (<1)
O1	0.77410 (13)	0.30501 (7)	0.43628 (10)	0.0642 (3)	
O2	0.56045 (16)	0.31504 (8)	0.29564 (11)	0.0795 (4)	
C1	0.65706 (18)	0.35245 (10)	0.36678 (13)	0.0540 (4)	
C2	0.66645 (18)	0.45392 (10)	0.38934 (13)	0.0529 (4)	
C3	0.5646 (2)	0.51221 (12)	0.31812 (17)	0.0750 (5)	
H3	0.4919	0.4871	0.2574	0.09*	
C4	0.5690 (3)	0.60709 (14)	0.3358 (2)	0.0901 (6)	
H4	0.5004	0.6458	0.2865	0.108*	
C5	0.6720 (3)	0.64395 (12)	0.4237 (3)	0.0912 (7)	
H5	0.6748	0.7082	0.4353	0.109*	
C6	0.7731 (3)	0.58741 (14)	0.4966 (2)	0.0971 (7)	
H6	0.8437	0.6134	0.5579	0.116*	
C7	0.7711 (2)	0.49201 (12)	0.47963 (18)	0.0738 (5)	
H7	0.8402	0.4537	0.5292	0.089*	
C8	0.77716 (18)	0.20698 (10)	0.42443 (13)	0.0552 (4)	
C9	0.8731 (2)	0.16674 (11)	0.34018 (14)	0.0623 (4)	
C10	0.8778 (2)	0.06901 (12)	0.33938 (17)	0.0732 (5)	
H10	0.9403	0.0392	0.2831	0.088*	
C11	0.7940 (2)	0.01520 (12)	0.41818 (18)	0.0759 (5)	
C12	0.7035 (2)	0.05916 (12)	0.50064 (19)	0.0786 (5)	
H12	0.6473	0.0239	0.5555	0.094*	
C13	0.6936 (2)	0.15527 (12)	0.50441 (16)	0.0682 (4)	
H13	0.6306	0.1845	0.5609	0.082*	
C14	0.9689 (3)	0.22412 (17)	0.2560 (2)	0.0964 (6)	
H14A	0.9884	0.1881	0.1854	0.145*	0.56 (3)
H14B	1.0745	0.2423	0.2958	0.145*	0.56 (3)
H14C	0.9052	0.2789	0.2328	0.145*	0.56 (3)
H14D	0.9904	0.2847	0.2906	0.145*	0.44 (3)
H14E	0.9042	0.2305	0.1802	0.145*	0.44 (3)
H14F	1.0735	0.1939	0.2432	0.145*	0.44 (3)
C15	0.8030 (3)	−0.09032 (13)	0.4133 (3)	0.1179 (10)	
H15A	0.771	−0.1159	0.4884	0.177*	0.67 (3)
H15B	0.9157	−0.1092	0.4002	0.177*	0.67 (3)
H15C	0.7279	−0.1128	0.3484	0.177*	0.67 (3)
H15D	0.8387	−0.1094	0.3362	0.177*	0.33 (3)
H15E	0.6941	−0.1161	0.4244	0.177*	0.33 (3)
H15F	0.8818	−0.1124	0.4762	0.177*	0.33 (3)

### Atomic displacement parameters (Å^2^)

**Table:**

	*U*^11^	*U*^22^	*U*^33^	*U*^12^	*U*^13^	*U*^23^
O1	0.0729 (7)	0.0412 (5)	0.0751 (7)	0.0067 (5)	−0.0183 (5)	−0.0071 (5)
O2	0.0873 (8)	0.0582 (7)	0.0878 (8)	0.0055 (6)	−0.0314 (7)	−0.0134 (6)
C1	0.0571 (8)	0.0488 (8)	0.0553 (8)	0.0039 (6)	−0.0018 (7)	−0.0026 (6)
C2	0.0542 (8)	0.0450 (7)	0.0601 (8)	0.0017 (6)	0.0072 (6)	0.0017 (6)
C3	0.0791 (11)	0.0585 (10)	0.0857 (12)	0.0119 (8)	−0.0066 (9)	0.0087 (8)
C4	0.0900 (14)	0.0574 (11)	0.1237 (17)	0.0170 (10)	0.0124 (13)	0.0235 (11)
C5	0.0875 (13)	0.0391 (9)	0.151 (2)	−0.0004 (9)	0.0357 (14)	0.0035 (11)
C6	0.1008 (15)	0.0578 (11)	0.1305 (18)	−0.0126 (10)	−0.0065 (13)	−0.0219 (11)
C7	0.0802 (11)	0.0480 (9)	0.0906 (12)	−0.0014 (8)	−0.0120 (9)	−0.0058 (8)
C8	0.0599 (9)	0.0420 (7)	0.0614 (9)	0.0049 (6)	−0.0113 (7)	−0.0057 (6)
C9	0.0636 (9)	0.0620 (9)	0.0598 (9)	0.0067 (7)	−0.0077 (7)	−0.0058 (7)
C10	0.0728 (11)	0.0665 (10)	0.0775 (11)	0.0210 (8)	−0.0152 (8)	−0.0246 (8)
C11	0.0760 (11)	0.0466 (9)	0.0998 (13)	0.0052 (7)	−0.0321 (9)	−0.0061 (8)
C12	0.0782 (12)	0.0592 (10)	0.0962 (13)	−0.0084 (8)	−0.0077 (9)	0.0118 (9)
C13	0.0691 (10)	0.0601 (10)	0.0752 (11)	0.0040 (8)	0.0039 (8)	−0.0023 (8)
C14	0.1010 (15)	0.1049 (16)	0.0852 (13)	0.0047 (12)	0.0189 (11)	0.0038 (12)
C15	0.1271 (19)	0.0463 (10)	0.170 (2)	0.0118 (10)	−0.0637 (18)	−0.0131 (12)

### Geometric parameters (Å, °)

**Table:**

O1—C1	1.3519 (17)	C10—C11	1.374 (3)
O1—C8	1.4108 (17)	C10—H10	0.93
O2—C1	1.1913 (17)	C11—C12	1.359 (3)
C1—C2	1.476 (2)	C11—C15	1.514 (2)
C2—C7	1.371 (2)	C12—C13	1.380 (2)
C2—C3	1.376 (2)	C12—H12	0.93
C3—C4	1.373 (3)	C13—H13	0.93
C3—H3	0.93	C14—H14A	0.96
C4—C5	1.339 (3)	C14—H14B	0.96
C4—H4	0.93	C14—H14C	0.96
C5—C6	1.367 (3)	C14—H14D	0.96
C5—H5	0.93	C14—H14E	0.96
C6—C7	1.379 (3)	C14—H14F	0.96
C6—H6	0.93	C15—H15A	0.96
C7—H7	0.93	C15—H15B	0.96
C8—C13	1.365 (2)	C15—H15C	0.96
C8—C9	1.376 (2)	C15—H15D	0.96
C9—C10	1.401 (2)	C15—H15E	0.96
C9—C14	1.494 (3)	C15—H15F	0.96
			
C1—O1—C8	117.59 (11)	C9—C14—H14B	109.5
O2—C1—O1	122.74 (14)	H14A—C14—H14B	109.5
O2—C1—C2	125.32 (14)	C9—C14—H14C	109.5
O1—C1—C2	111.93 (12)	H14A—C14—H14C	109.5
C7—C2—C3	118.87 (15)	H14B—C14—H14C	109.5
C7—C2—C1	122.47 (14)	C9—C14—H14D	109.5
C3—C2—C1	118.65 (14)	H14A—C14—H14D	141.1
C4—C3—C2	120.70 (19)	H14B—C14—H14D	56.3
C4—C3—H3	119.7	H14C—C14—H14D	56.3
C2—C3—H3	119.7	C9—C14—H14E	109.5
C5—C4—C3	120.17 (19)	H14A—C14—H14E	56.3
C5—C4—H4	119.9	H14B—C14—H14E	141.1
C3—C4—H4	119.9	H14C—C14—H14E	56.3
C4—C5—C6	120.23 (17)	H14D—C14—H14E	109.5
C4—C5—H5	119.9	C9—C14—H14F	109.5
C6—C5—H5	119.9	H14A—C14—H14F	56.3
C5—C6—C7	120.4 (2)	H14B—C14—H14F	56.3
C5—C6—H6	119.8	H14C—C14—H14F	141.1
C7—C6—H6	119.8	H14D—C14—H14F	109.5
C2—C7—C6	119.64 (18)	H14E—C14—H14F	109.5
C2—C7—H7	120.2	C11—C15—H15A	109.5
C6—C7—H7	120.2	C11—C15—H15B	109.5
C13—C8—C9	122.33 (15)	H15A—C15—H15B	109.5
C13—C8—O1	117.84 (14)	C11—C15—H15C	109.5
C9—C8—O1	119.64 (14)	H15A—C15—H15C	109.5
C8—C9—C10	116.07 (16)	H15B—C15—H15C	109.5
C8—C9—C14	121.84 (16)	C11—C15—H15D	109.5
C10—C9—C14	122.09 (17)	H15A—C15—H15D	141.1
C11—C10—C9	122.86 (16)	H15B—C15—H15D	56.3
C11—C10—H10	118.6	H15C—C15—H15D	56.3
C9—C10—H10	118.6	C11—C15—H15E	109.5
C12—C11—C10	118.26 (16)	H15A—C15—H15E	56.3
C12—C11—C15	121.0 (2)	H15B—C15—H15E	141.1
C10—C11—C15	120.7 (2)	H15C—C15—H15E	56.3
C11—C12—C13	121.14 (18)	H15D—C15—H15E	109.5
C11—C12—H12	119.4	C11—C15—H15F	109.5
C13—C12—H12	119.4	H15A—C15—H15F	56.3
C8—C13—C12	119.34 (17)	H15B—C15—H15F	56.3
C8—C13—H13	120.3	H15C—C15—H15F	141.1
C12—C13—H13	120.3	H15D—C15—H15F	109.5
C9—C14—H14A	109.5	H15E—C15—H15F	109.5
			
C8—O1—C1—O2	−1.4 (2)	C1—O1—C8—C9	89.23 (17)
C8—O1—C1—C2	179.25 (12)	C13—C8—C9—C10	1.2 (2)
O2—C1—C2—C7	174.36 (17)	O1—C8—C9—C10	176.08 (13)
O1—C1—C2—C7	−6.4 (2)	C13—C8—C9—C14	−177.81 (16)
O2—C1—C2—C3	−4.7 (2)	O1—C8—C9—C14	−2.9 (2)
O1—C1—C2—C3	174.61 (14)	C8—C9—C10—C11	−0.7 (2)
C7—C2—C3—C4	0.9 (3)	C14—C9—C10—C11	178.30 (17)
C1—C2—C3—C4	−179.98 (17)	C9—C10—C11—C12	−0.3 (3)
C2—C3—C4—C5	−0.6 (3)	C9—C10—C11—C15	179.91 (16)
C3—C4—C5—C6	−0.1 (3)	C10—C11—C12—C13	0.9 (3)
C4—C5—C6—C7	0.5 (3)	C15—C11—C12—C13	−179.29 (17)
C3—C2—C7—C6	−0.5 (3)	C9—C8—C13—C12	−0.7 (2)
C1—C2—C7—C6	−179.57 (18)	O1—C8—C13—C12	−175.61 (14)
C5—C6—C7—C2	−0.2 (3)	C11—C12—C13—C8	−0.5 (3)
C1—O1—C8—C13	−95.67 (17)		
